# Impact of Faba Bean-Seed Rhizobial Inoculation on Microbial Activity in the Rhizosphere Soil during Growing Season

**DOI:** 10.3390/ijms17050784

**Published:** 2016-05-20

**Authors:** Anna Siczek, Jerzy Lipiec

**Affiliations:** Institute of Agrophysics, Polish Academy of Sciences, Department of Soil and Plant System, P.O. Box 201, 20-290 Lublin, Poland; j.lipiec@ipan.lublin.pl

**Keywords:** soil enzymes, rhizosphere, *Vicia faba*, functional diversity of soil, *Rhizobium leguminosarum*, biofertilizers

## Abstract

Inoculation of legume seeds with *Rhizobium* affects soil microbial community and processes, especially in the rhizosphere. This study aimed at assessing the effect of *Rhizobium* inoculation on microbial activity in the faba bean rhizosphere during the growing season in a field experiment on a Haplic Luvisol derived from loess. Faba bean (*Vicia faba* L.) seeds were non-inoculated (NI) or inoculated (I) with *Rhizobium leguminosarum* bv. *viciae* and sown. The rhizosphere soil was analyzed for the enzymatic activities of dehydrogenases, urease, protease and acid phosphomonoesterase, and functional diversity (catabolic potential) using the Average Well Color Development, Shannon-Weaver, and Richness indices following the community level physiological profiling from Biolog EcoPlate™. The analyses were done on three occasions corresponding to the growth stages of: 5–6 leaf, flowering, and pod formation. The enzymatic activities were higher in I than NI (*p* < 0.05) throughout the growing season. However, none of the functional diversity indices differed significantly under both treatments, regardless of the growth stage. This work showed that the functional diversity of the microbial communities was a less sensitive tool than enzyme activities in assessment of rhizobial inoculation effects on rhizosphere microbial activity.

## 1. Introduction

Gram-negative soil bacteria of the genus *Rhizobium* play a very important role in agriculture. They form a symbiotic relationship with leguminous crops resulting in biological nitrogen fixation and thereby reduction of the requirements for added nitrogenous fertilizer during the growing season. *Rhizobium* symbiosis with legumes produces 50% of 175 million tons of total biological N2 fixation annually worldwide [1]. Therefore, inoculation of legumes with efficient rhizobia at sowing is one of the most important and agronomically eco-friendly practices used for improvement of N fixation. Denton *et al.* [2] observed that nodule dry matter increased in soil with an increasing rate of inoculation. Besides enhanced nodulation and nitrogen fixation, rhizobial seed inoculation can stimulate production of phytohormones, siderophores, and HCN (hydrogen cyanide) as well as microbial diversity and structure, potentially enhancing plant growth-promoting rhizobacteria [3,4]. Rhizobial inoculation (with *R. leguminosarum*) enhanced phosphate solubilization [1,5] as well as P and N uptake and Fe content in lettuce and carrots [6]. Inoculation with a *R. gallicum* strain induced growth of bacterial communities that had been often reported as PGPM (plant growth-promoting microorganisms) [3]. The extent of these changes was also seen in the next rotation crop. Therefore, rhizobial inoculation resulted in increased shoot growth, number of pods, and grain yield of faba bean [2] and lentil [5], compared with non-inoculated controls.

Using T-RFLP (Terminal Restriction Fragment Length Polymorphism) analysis, Trabelsi *et al.* [4] showed that inoculation of common bean with rhizobial strains resulted in a highly significant increase in the TRF (Terminal Restriction Fragments) number (51 TRFs), compared to non-inoculated plants (13 TRFs). Additionally, among the total number of 84 TRFs, 23 were specifically induced by inoculation, indicating that inoculation with rhizobia increased microbial diversity and structure.

Rhizobia can also serve as biological control agents of some plant pathogens in legume and non-legume plants [7]. In fields infested with *Pythium* spp., inoculation with *Rhizobium leguminosarum* significantly increased seedling emergence in pea and sugar beets [8] and reduced the incidence of a disease induced by soil-borne pathogens of pea and lentil [9]. Treatment of faba bean seeds with *R. leguminosarum* under soil infestation conditions by *R.* *solani* caused significant reduction in damping-off as compared to the untreated control [10].

Rhizobial seed inoculation may have a significant influence on the growth and composition of the microbiome as well as associated synthesis and release of enzymes into soil, especially in the rhizosphere. The rhizosphere is a narrow zone of soil that is influenced by root exudates and inhabited by most microorganisms (bacteria and fungi), including those beneficially affecting soil health and ecosystem functioning [11]. Rhizobacteria with growth-promoting and pathogen-suppressive capability were isolated from healthy chickpea (*Cicer arietinum* L.) plants and identified as *Pseudomonas* and *Bacillus* [12].

Little information is available about the effect of *Rhizobium* inoculation on the soil microbial activity in the faba bean rhizosphere. Faba bean is an important leguminous crop. It occupies *ca.* 5.3% of the sown area of fodder legumes in Poland [13]. The objectives of this study were (1) to determine the effect of inoculation of faba bean seeds with *Rhizobium* on soil microbial activity; and (2) to compare the activity of actual soil microbial community using enzyme activities with the functional diversity of the cultivable community using Biolog Ecoplates of field soil with low resident rhizobia. To date, according to our knowledge, no studies with faba bean concerning the microbial effect of inoculation with rhizobia were conducted using both approaches simultaneously.

## 2. Results and Discussion

### 2.1. Enzyme Activities

**Figure 1:**
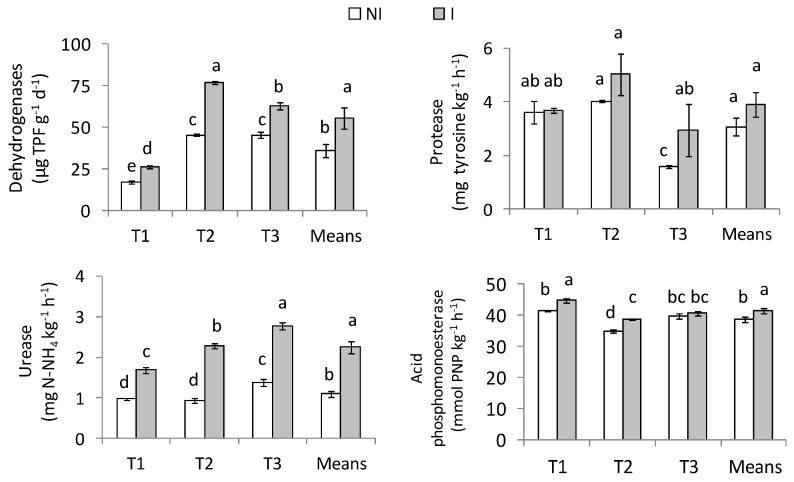
Enzymatic activity. Different lowercase letters indicate significant differences (*p* < 0.05). NI, non-inoculated; I, inoculated with *Rhizobium*; T1, 5–6 leaf stage; T2, flowering; T3, pod formation stage.

All enzymatic activities (except for protease activity at T1 and T2 and acid phosphomonoesterase at T3) were higher in inoculated (I) than non-inoculated (NI) (*p* < 0.05) throughout the growing season although the extent of the differentiation was related to the type of the microbial activity and measurement occasion (Figure 1). At T1 and T2 (5–6 leaf and flowering stages, respectively), the relative differences between the two treatments were appreciably higher in urease activity (72%–143%) and dehydrogenase activity (55%–70%) than in the activities of acid phosphomonoesterase (8%–11%) and protease (3%–25%), whereas at T3 the differences amounted to 86% in protease and 102% in urease activities.

When annual averages were taken into consideration, all the evaluated enzymatic activities were improved by the inoculation of the seeds with rhizobia. The most pronounced improvement produced by the inoculation was noticed for urease (104%), while acid phosphomonoesterase activity was changed to the lowest extent (7%). However, this increase for protease was insignificant. The above results indicate that the enzymatic activities in our study were not similarly affected by the *Rhizobium* inoculation during the growing season. The relatively high sensitivity of urease activity and dehydrogenase activity at growth stages T1 (5–6 leaf) and T2 (flowering) indicate that *Rhizobium* inoculation has great potential for the cycling of N and production of adenosine triphosphate through oxidation of organic matter in the soil, respectively. However, the less pronounced increases in the activities of acid phosphomonoesterase and protease indicate respectively low release of both inorganic phosphorus (orthophosphate) from organic phosphomonesters [14] and protein N at the hydrolysis [15,16].

The changes in a majority of the enzymatic activities between the measurement occasions were statistically different (*p* < 0.05) both in NI and I treatments. Irrespective of the treatment, maximum values of dehydrogenase and protease activities were noted at T2, urease activity at T3, and the activity of acid phosphomonoesterase at T1. The maximum values of dehydrogenase and protease activities at T2 (flowering) can be linked to abundant root size and organic carbon from root exudates in the rhizosphere at this growth stage and hence increasing microbial populations contributing to release of these enzymes [11]. This effect was more pronounced in I than NI. The *Rhizobium* inoculation and enhanced enzymatic activity may improve plant growth through soil nutrient enrichment and increase of resistance against plant pathogens [17].

### 2.2. Community Level Physiological Profiling (CLPP)

AWCD (average well-color development) was higher in T2 than T1 and T3 under both treatments (Table 1). However, the differences between both treatments were not significant, regardless of the sampling time. Similarly, annual averages did not differ after the rhizobial inoculation. The R index did not differ between the NI and I treatments in all sampling terms and for the annual mean. However, it decreased significantly in T3, in comparison with T2 under I. Although H did not differ between both treatments in individual sampling terms, it significantly increased under I in T3, compared to T1 and T2. The mean value of H was higher under I than NI.

**Figure 2:**
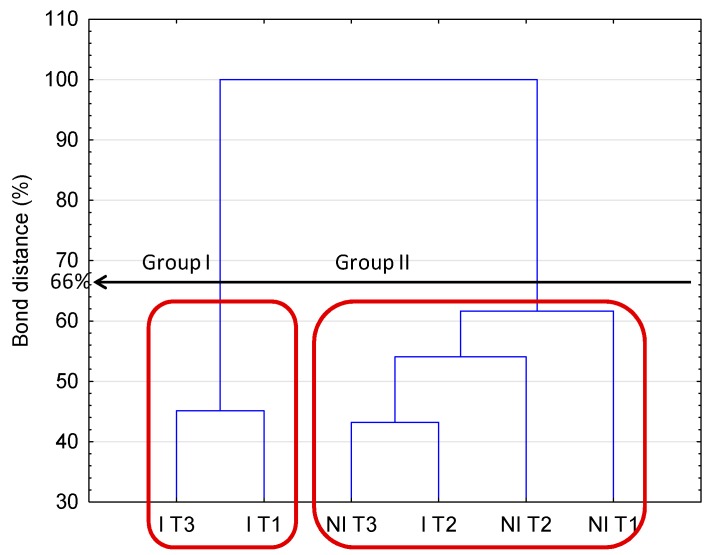
Dendrogram of the bond distances between the carbon utilizations patterns of the substrates on the Biolog EcoPlates^TM^. Grouping was conducted according to the Sneath’s criterion (66%); NI, non-inoculated; I, inoculated with *Rhizobium*; T1, 5–6 leaf stage; T2, flowering; T3, pod formation stage; *n* = 3. Red frames indicate group treatments with similar carbon utilization.

**Figure 3:**
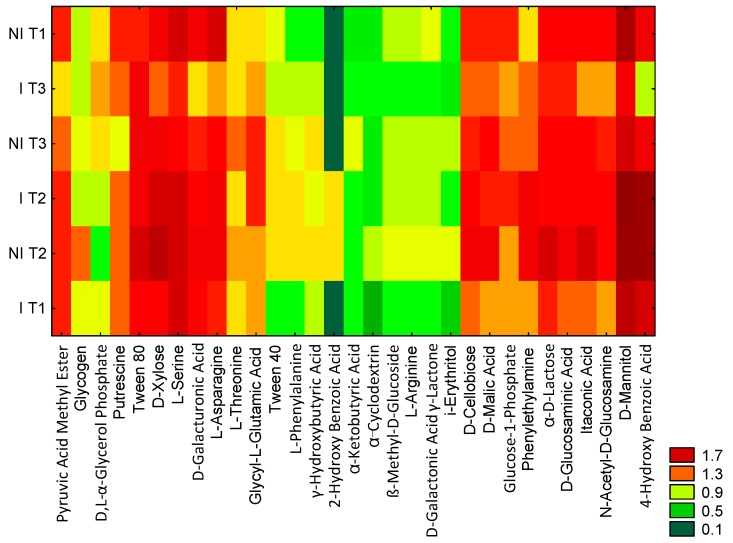
Biolog EcoPlate^TM^ carbon substrates utilization intensity diagram. NI, non-inoculated; I, inoculated with *Rhizobium*; T1, 5–6 leaf stage; T2, flowering; T3, pod formation stage; *n* = 3.

**Figure 4:**
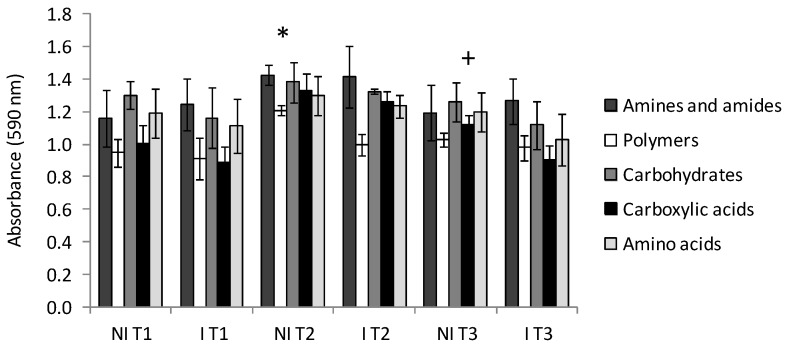
Utilization of categorized substrate by the microbial communities. Errors bars indicate the standard deviations of the mean; * indicates significant difference in polymers utilization between NI T2 and I T2; and + indicates difference in carboxylic acids utilization between NI T3 and I T3 (*p* < 0.05); *n* = 3. NI, non-inoculated; I, inoculated with *Rhizobium*; T1, 5–6 leaf stage; T2, flowering; T3, pod formation stage.

Figure 2 presents the bond distances between the treatments, where clustering was evaluated using the carbon utilization patterns of the substrates. Taking into consideration Sneath’s criterion 66%, two groups were distinguished. A similar response was noted under I T3 and I T1 (group 1) in terms of carbon substrate utilization. These findings were supported by the carbon substrate utilization intensity (Figure 3). A lower degree of substrate utilization was observed in group 1 than 2. The lower rate of utilization of substrates by group 1 than 2 was found for *N*-Acetyl-d-Glucosamine, d-Galactonic Acid γ-Lactone, l-Asparagine, d-Cellobiose, α-d-Lactose, d-Malic Acid, l-Arginine, β-Methyl-d-Glucoside, and Itaconic Acid. Significant differences among the treatments were observed in the utilization patterns of categorized substrates (Figure 4). The highest differences between the NI and I treatment were noted for polymers in T2 and carboxylic acids in T3 when both categorized substrates were used to a lesser extent under I than NI.

Analyses of variance (Table 2) showed that the functional diversity of the microbial communities was a less sensitive tool for assessment of rhizobial inoculation effects than soil enzyme activities, indicating that soil enzyme activities may reveal more about microbial activity of soil. This may be related to the fact that CLPP could select rare, less-dominant, but culturable members of the community that have adapted to rapid growth on available substrates. As opposed to the CLPP, soil enzyme activity is a cultivation-independent method and can reflect the functioning of the microbial community. It is worth noting that soil enzyme activities (Figure 1), compared to the functional diversity via Biolog EcoPlates (Table 1), were more sensitive to the rhizobial inoculation throughout the growing season. This result is in agreement with earlier findings indicating that the Biolog method can be suitable for detecting changes in substrate availability for microorganisms shortly after soil modification than during a later period [18].

Overall, the results from the present study on the enzyme activity and functional diversity of a rhizosphere environment agree well with earlier results indicating that root-mediated processes enhance soil aggregation and plant drought resistance and affect soil acidity and accessibility of nutrients to plants [11].

## 3. Materials and Methods

### 3.1. Description of the Study Site and Treatments

The field study was performed in Lublin, Poland (51°15′ N, 22°35′ E). The soil was a Haplic Luvisol [19] derived from loess, with clay, silt, and sand contents of 70, 290, and 640 g·kg^−1^, respectively, in the 0–20 cm soil layer, pH 6.1 (H_2_O), and organic matter content of 14.1 g·kg^−1^. As indicated by the Kjeldahl method, total N was 0.75 g·kg^−1^, and P, K, Mg contents were 114, 153, and 39 mg·kg^−1^, respectively. The soil was under 30-year conventional tillage, with main tillage operations including pre-plow (10 cm depth) + harrowing and moldboard plowing (20–25 cm depth). Faba bean (*Vicia faba* L.) cultivar Granit was used as the test crop. Faba bean seeds were non-inoculated (NI) or inoculated (I) with commercial inoculum of *Rhizobium leguminosarum* bv. *viciae* on perlite (Nitragina) obtained from the Institute of Soil Science and Plant Cultivation (IUNG). The number of *Rhizobium* was 10^6^ CFU·g^−1^ perlite. The inoculum was applied immediately before sowing, in accordance with the recommendations of the manufacturer. The plots (2 m × 2 m) were randomly organized in three replicates.

### 3.2. Sampling of Rhizosphere Soil

Faba bean roots were excavated from the 0–15 cm soil layer and shaken gently to separate loosely adhering soil. The soil left adhering to the roots (rhizosphere soil) was vigorously shaken and taken for further analysis. Soil for microbial analyses was taken three times during the vegetative period of faba bean: at the T1 5–6 leaf, T2 flowering, and T3 pod formation stages. Soil was passed through a 0.2 cm mesh sieve and used immediately for analyses or it was short-term stored at 4 °C.

### 3.3. Enzymatic Activities

Dehydrogenase activity was determined using the Thalmann [20] method, modified by Alef [21], after soil incubation with 2,3,5-triphenyltetrazolium chloride (TTC) as a substrate. Triphenyl formazan (TPF) absorbance was measured at 485 nm. Urease activity was assessed with urea solution as a substrate according to Zantua and Bremner [22] method. The Ladd and Butler [23] method modified by Alef and Nannipieri [24] was used for protease activity measurement. The concentration of tyrosine in soil after one-hour incubation at 50 °C with a TRIS-HCl (pH 8.1) sodium caseinate solution was measured at 578 nm. Acid phosphomonoesterase was determined by the Tabatabai and Bremner [25] method after soil incubation with *p*-nitrophenyl disodium phosphate and by measuring the *p*-nitrophenol (PNP) concentration at 400 nm. Four replicates per treatment were done for each analysis. Results were calculated in reference to oven-dry (105 °C) weight of soil.

### 3.4. Community Level Physiological Profiling

Community Level Physiological Profiling was evaluated using Biolog EcoPlate™ (Biolog Inc., Hayward, CA, USA) with 31 carbon sources [26]. Each well of the Biolog EcoPlate™ was inoculated with 120 μL of the inoculum and incubated at 27 °C. Absorbance data were taken every 24 h for 72 h at 590 nm using a plate reader Biolog MicroStation™. On the basis of data obtained at 72 h, Richness (R), Shannon-Weaver (H), and average well color development (AWCD) indices were calculated following Garland and Millis [27].

### 3.5. Statistical Analysis

Statistical analyses were performed with Statistica 10.0 software (StatSoft Inc., Tulsa, OK, USA, 2011). Collected data were subjected to two-way analysis of variance (ANOVA) for comparing means, and significant differences were calculated with post-hoc Tukey’s HSD (honestly significant differences) test at a *p* < 0.05 significant level. Cluster analysis, including grouping of treatments and features, was conducted on standardized data from the average absorbance results at 72 h. To indicate the similarity of the carbon utilization patterns of the substrates on the Biolog EcoPlate^TM^ between the treatments, a dendrogram was prepared with scaled bond distances on the axis (method of Ward) and boundary marked according to Sneath’s criterion (66%). The results were standardized according to AWCD in each microplate in order to remove the inoculum density effects [28].

## 4. Conclusions

This work showed that *Rhizobium* inoculation induced a significant and consistent increase in a majority of enzymatic activities in the rhizosphere throughout the vegetative period of faba bean. *Rhizobium* inoculation can affect selectively and variously the enzymatic activity depending on the enzyme type and plant growth stage. The functional diversity of microbial communities determined using Biolog EcoPlates is a less sensitive tool than enzyme activities in assessment of rhizobial inoculation effects on rhizosphere microbial activity.

## Figures and Tables

**Table 1:** Community Level Physiological Profiling (CLPP) indices.

Time	Treatment	CLPP		
		AWCD	R	H
T1	NI	1.13 a	30.0 ab	3.376 b
	I	1.15 a	30.0 ab	3.391 b
T2	NI	1.32 a	30.7 ab	3.394 ab
	I	1.25 a	31.0 a	3.392 b
T3	NI	1.17 a	30.3 ab	3.398 ab
	I	1.08 a	29.7 b	3.419 a
Means	NI	1.21 a	30.2 a	3.389 b
	I	1.16 a	30.3 a	3.401 a

Different lowercase letters within the same variables mean significant differences (*p* < 0.05). AWCD, average well-color development; R, richness index; H, Shannon-Weaver index; NI, non-inoculated; I, inoculated with *Rhizobium*; T1, 5–6 leaf stage; T2, flowering; T3, pod formation stage.

**Table 2:** *F*-values for enzyme activities and Community Level Physiological Profiling (CLPP) indices.

Factors	Enzyme Activities				CLPP		
	Dehydrogenases (µg TPF g^−1^·d^−1^)	Urease (mg N-NH_4_ kg^−1^·h^−1^)	Protease (mg Tyrosine kg^−1^·h^−1^)	Acid Phosphomonoesterase (mmol PNP kg^−1^·h^−1^)	AWCD	R	H
Inoculation	496.378 ***	406.903 ***	3.3857	34.9 ***	1.233	0.33	7.09 *
Time	316.912 ***	57.686 ***	8.8197 **	60.65 ***	6.047 *	8.33 **	10.39 **
Inoculation*Time	35.981 ***	15.03 ***	0.7304	4.05 *	0.521	2.33	2.46

AWCD, average well-color development; R, richness; H, Shannon-Weaver index; Probability at * *p* < 0.05, ** *p* < 0.01, *** *p* < 0.001.
